# Expression of Inflammation Depending on the Stage of Cervical Cancer

**DOI:** 10.3390/medicina60030349

**Published:** 2024-02-20

**Authors:** Agne Vitkauskaite, Daiva Urboniene, Joana Celiesiute, Kristina Jariene, Saulius Paskauskas, Daiva Vaitkiene, Astra Vitkauskiene

**Affiliations:** 1 Department of Obstetrics and Gynaecology, Medical Academy, Lithuanian University of Health Sciences, Eiveniu Str. 2, LT-50161 Kaunas, Lithuania; agne.vitkauskaite@lsmu.lt (A.V.); joana.celiesiute@lsmu.lt (J.C.); kristina.jariene@lsmu.lt (K.J.); saulius.paskauskas@lsmu.lt (S.P.); daiva.vaitkiene@lsmu.lt (D.V.); 2 Department of Laboratory Medicine, Medical Academy, Lithuanian University of Health Sciences, Eiveniu Str. 2, LT-50161 Kaunas, Lithuania; daiva.urboniene@lsmu.lt

**Keywords:** cervical cancer, inflammatory markers, interleukin-6, lipocalin-2, stages of cervical cancer, TREM-1

## Abstract

*Background and Objectives:* Cervical cancer (CC) remains a major public health problem, ranking as the fourth most common cause of cancer incidence and mortality in women globally. The development of CC is believed to be closely related to chronic inflammation. Thus, we aimed to evaluate the expression of systemic inflammation in patients with CC and to determine the threshold prognostic value of the systemic inflammation markers for CC and its advanced stage. *Materials and Methods:* 182 participants were recruited: 94 histology-proven patient with CC and 88 healthy women with NILM confirmed by liquid-based cytology test. The pre-treatment serum concentrations of cytokines, including IFN-β, IFN-γ, IL-1β, IL-2, IL-6, IL-10, IL-12p70, LCN2, TREM-1, and TNF-α, were determined for all study patients. *Results:* The odds ratio (OR) of having IL-6 concentration >17.4 pg/mL in the CC group compared to control patients was 11.4 (95% CI: 4.897–26.684); that of having TREM-1 concentration >355.6 pg/mL was 5.9 (95% CI: 2.257–15.767); and that of having LCN2 concentration >23,721.5 pg/mL was 3.4 (95% CI: 1.455–8.166). The odds ratio (OR) of having IL-6 concentration >28.7 pg/mL in advanced-stage CC (III–IV stage) compared to early-stage CC (I–II stage) was 2.921 (95% CI: 1.06–8.045), and that of having LCN2 concentration >25,640.0 pg/mL was 4.815 (95% CI: 1.78–13.026). *Conclusions:* The pre-treatment serum inflammation markers IL-6, TREM-1, and LCN2 at specified levels could be used as predictors of cervical cancer, and IL-6 and LCN2 as predictors of an increased chance of advanced-stage (III–IV stages) cervical cancer. Patients with cervical cancer had expressed systemic inflammation, and expression of inflammation elevated the chance of having CC and advanced-stage disease.

## 1. Introduction

Cervical cancer (CC) remains a major public health problem, ranking as the fourth most common cause of cancer incidence and mortality in women globally [1]. CC is the most commonly diagnosed cancer in 23 countries worldwide and the leading cause of cancer-related death in 36 countries worldwide [2]. Worldwide, an estimated 604,000 new cases and about 342,000 cervical-cancer-related deaths occurred in 2020 [1]. According to the European Cancer Information System (ECIS) report, CC was estimated to be the 13th most diagnosed cancer and the 9th leading cause of cancer-related death in the EU in 2022. Lithuania was ranked sixth among EU countries in terms of incidence (18.3 cases per 100,000 women) and seventh place in terms of mortality (11.0 cases per 100,000) in 2022 [3].

The development of CC is closely related to chronic inflammation, in some cases caused by high-risk human papillomavirus (HR-HPV)—a known trigger of the inflammation [4]. The relationship between inflammation, innate immunity, and cancer has been widely accepted recently, and scientists have focused on inflammatory markers in various malignant diseases [5,6].

Recent studies have shown that the evaluation of inflammation is easily available and is useful as an informative and cost-effective method to identify patients at high risk of inflammation-related disease development, progression, or recurrence. The value of common conventional inflammatory markers (e.g., C-reactive protein (CRP), white blood cells (WBC), erythrocyte sedimentation rate (ESR) and various ratios of inflammation-related cells, derived from hematology measurements) as prognostic factors has been assessed in various gynecological human cancers—cervical, endometrial, uterine, vulvar, and ovarian cancer [7,8,9,10,11]. Li et al. found out that ratios of inflammation-related cells (e.g., Neutrophil-to-Lymphocytes Ratio (NLR) and Monocyte-to-Lymphocytes Ratio (MLR)) were independent prognostic factors as well as risk factors for recurrence in patients with stage IIB of CC. Li et al. determined that NLR could be used as a potential marker for therapeutic response in CC [7]. Holub et al. concluded that lymphopenia and lymphocytes-related ratios were associated with a poorer outcome in patients with endometrial cancer classified as high-risk and treated with adjuvant with postoperative external beam radiotherapy and could be considered at cancer diagnosis [7]. Winarto et al. demonstrated that pre-treatment inflammation markers indicate an essential association with adverse clinical outcomes of vulvar cancer. It was proven that the unique values of examined inflammatory markers at particular cut-offs had been tailored for specific endpoints [10].

Most recent cancer studies focused on the above-mentioned conventional routine inflammatory markers. However, studies investigating the role of serum soluble mediators (e.g., cytokines, etc.) as more modern inflammatory markers to determine whether these pre-treatment inflammatory markers can be used as associative markers for the disease prognosis and staging are lacking. We aimed to provide new data on the relevance of inflammatory biomarkers as prognostic indicators of CC to evaluate the expression of systemic inflammation in patients with CC and to determine the threshold prognostic value of the systemic inflammation markers for CC and its advanced stage.

## 2. Materials and Methods

### 2.1. Patients and Study Design

This study was carried out on 94 patients with histology-proven CC. All patients with CC had newly diagnosed Cervical Squamous-Cell Carcinoma. Patients with cervical cancer other than Cervical Squamous-Cell Carcinoma were excluded from the study. Cancer staging was determined based on the International Federation of Gynaecology and Obstetrics (FIGO) 2021 revised classification system [4]. The control group consisted of 88 healthy women with a cervico-vaginal cytology test negative for intraepithelial lesions or malignancy (NILM), confirmed by liquid-based cytology test (SurePath, Becton Dickinson, Franklin Lakes, NJ, USA) and reported according to the 2014 Bethesda System for reporting cervical cytology [12]. 

The exclusion criteria for all study participants were as follows: women with any autoimmune diseases, active or chronic infections, cardiovascular diseases, connective tissue diseases, diabetes, and pregnancy, a history of malignant tumors, and younger than 18 years. None of the patients previously received immunosuppressive treatment, radiotherapy, chemotherapy, or had previously received treatment for carcinoma in situ or HSIL.

HPV status was assessed for all patients using PCR-based Multiplex HPV genotyping (Optiplex HPV Genotyping Kit, Diamex, Heidelberg, Germany) for detection and differentiation of HPV 24 most common genotypes in CC samples.

All women were treated in the Department of Obstetrics and Gynaecology of Hospital of Lithuanian University of Health Sciences Kauno Klinikos. The study was approved by the Kaunas Regional Biomedical Research Ethics Committee (No. BE-2-80/2018), and written consent from all subjects was received.

### 2.2. Determination of Inflammatory Markers Concentration

Venous blood samples were drawn from each study patient before any procedures, i.e., surgery and cancer treatment. Each blood sample was centrifuged, aliquoted, and stored at −80 °C until analyses. The serum concentrations of nine different cytokines, including IFN-β, IFN-γ, IL-1β, IL-2, IL-6, IL-10, IL-12p70, lipocalin-2 (LCN2), and triggering receptor expressed on myeloid cells 1 (TREM-1), were quantified via a magnetic bead-based multiplex assay (Human Cytokine Premixed Multi-Analyte Kit, R&D, Minneapolis, MN, USA) and a Luminex 100 Analyzer (Luminex Corp., Austin, TX, USA), according to manufacturer’s instructions. Each sample was analyzed in triplicate. Commercial ELISA kits (DIAsource, Louvain-la-Neuve, Belgium) were used to measure serum TNF-α levels.

### 2.3. Statistical Analysis

Statistical data analysis was performed using the statistical package IBM SPSS 23.0. In the case–control study, the selected ratio was 1:1 to match cases based on a single characteristic—diagnosis (presence vs. absence of CC). The Kolmogorov–Smirnov test was employed to determine distribution of quantitative data. For the analysis of normally distributed data, Student’s *t*-test was applied, and for the analysis of non-parametric data, the Mann–Whitney test was applied. The Chi-square test was used to determine whether a relationship exists between qualitative data. Differences comparing the groups were considered statistically significant when a *p* value was less than 0.05. The study power analysis was carried out and determined to be 0.8. Discrimination values were assessed using ROC and AUC. Analyzing the impact of a factor on disease development, the odds ratio (OR) was calculated. The factor was considered significant if the lower and upper limits of the OR 95% confidence interval (CI) were less than or greater than 1.

## 3. Results

A total of 182 women aged 22–91 years were included in the analysis. The patients with CC were significantly older compared to the NILM group (*p* = 0.004), but we used a random function to get an equal control group (Table 1).

The majority of women in the CC group were HPV-positive, and this percentage was significantly higher compared to the percentage of HPV-positive women in the control group (98.9% vs. 13.6%, *p* < 0.001). 

The study determined the threshold value of TNF-α, IFN-β, IFN-γ, IL-1β, IL-6, LCN2, and TREM-1 in relation to established cytological findings using the ROC curve method, which revealed the prognostic value of the studied inflammatory markers (Table 2).

Based on the data, the final multivariable model of binary logistic regression analysis for cervical cancer was predicted (Table 3). The odds ratio (OR) of having LCN2 concentration >23,721.5 pg/mL in the CC group compared to control patients was 3.4 (95% CI: 1.455–8.166); that of having TREM-1 concentration >355.6 pg/mL in the CC group compared to control patients was 5.9 (95% CI: 2.257–15.767); and that of having IL-6 concentration >17.4 pg/mL in the CC group compared to control patients was 11.4 (95% CI: 4.897–26.684).

During the study, the significant differences in IL-1β, IL-6, LCN2, and TREM-1 were found depending on the stage of the disease (Table 4). The values of these markers in the advanced-stage CC (III–IV stage) group were significantly higher compared to the early-stage CC (I–II stage) group. 

In our study, we determined the threshold value of four inflammatory markers (IL-1β, IL-6, LCN2, and TREM-1) according to the stage of CC in patients using the ROC curve method, but the prognostic value was revealed only for IL-1β, IL-6, and LCN2 (Table 5).

Based on the data, the final multivariable model of binary logistic regression analysis for advanced-stage (III–IV stage) cervical cancer was predicted (Table 6). The odds ratio (OR) of having LCN2 concentration >25,640.0 pg/mL in advanced-stage CC (III–IV stage) compared to early-stage CC (I–II stage) was 4.815 (95% CI: 1.78–13.026), and that of having IL-6 concentration >28.7 pg/mL in advanced-stage CC (III–IV stage) compared to early-stage CC (I–II stage) was 2.921 (95% CI: 1.06–8.045). 

## 4. Discussion

For the first time, the present study determined the threshold values and revealed the significant potential prognostic value of serum soluble mediators (serum cytokines): pre-treatment serum IL-6, TREM-1, LCN2—for CC, and IL-6, LCN2—for advanced-stage (III–IV stage) CC. The use of serum cytokines as prognostic indicators of CC not only provided information related to the presence of systemic inflammation in CC but allowed them to be used for prognostic purposes.

Inflammation might be called the secret killer due to its implication in many pathologies, including cancers. Cancer incidence rises with the presence and persistence of inflammation, reflected by increasing levels of inflammatory markers [5,6]. In various gynecological and non-gynecological human cancers, the conventional biochemical or hematological markers routinely measured in common blood tests or as ratios derived from these measurements (CRP, WBC, ESR, calculated inflammatory cell-derived indices or ratios) have been intensively assessed not only for confirmation of the presence of inflammation but also as prognostic factors [7,8,9,10,11]. The majority of the studies were able to confirm the relationship between inflammation and end-points of cancer diseases and to disclose the prognostic value of the analysis of CRP, NLR, MLR, and other ratios or indices of inflammation-related cells [7,8,9,10,11].

The other approach to inflammatory response evaluation could be analysis of various soluble mediators, e.g., cytokines, interleukins, etc. These markers are explored less. Some studies and publications have reported a relationship between dysregulation of expression of some cytokines, including IL-1, IL-2, IL-4, IL-6, LPC2, TNF-α, etc., and incidence of cancer, cancer invasivity, and metastases [13,14]. It has been accepted that cytokine-related immune responses in cancer are complex and incompletely understood due to cytokine functional duality. The ambiguity of immuno-stimulatory T-helper (TH) cell type 1 (TH1-type) cytokines, including TNF-α, IFN, IL-2, IL-12, etc., lies in the fact that they can induce cell-mediated immunity and tumor suppression; still, they can also act as pro-inflammatory players. Inhibitory TH2-type cytokines, including IL-4, IL-6, IL-8, IL-10, etc., also demonstrate dual functions: they can reduce cell-mediated immunity and induce humoral immunity [15]. Nevertheless, the lack of knowledge need not be a barrier to trying to apply these markers to cancer research. 

During this study, we focused on IL-6, a soluble mediator with a pleiotropic effect on inflammation and immune response, well known as one of the most important mediators of inflammation. We measured the serum pre-treatment levels of IL-6 in patients with CC and in controls and found that the serum IL-6 levels in patients with CC were markedly higher than those in controls. Various studies examined and evaluated IL-6′s significance in cancer initiation, metastasis, and development [14,16]. IL-6 is mainly produced by immune and non-immune cells, including T cells, monocytes, macrophages, and dendritic cells. Some studies reported IL-6 production by cancer cells, i.e., CC cells [17]. IL-6, as an essential trigger of epithelial-to-mesenchymal transition, activates STAT3, followed by transcription of ZEB1 and production of mesenchymal-like proteins and a mesenchymal phenotype, which is utilized by carcinoma cells to promote invasion, angiogenesis, metastasis, and apoptosis. [18,19]. The expression of IL-6 was shown to be higher in cancerous tissues than in adjacent noncancer tissues in patients with early-stage CC [20]. It was proved that after IL-6 is synthesized in a local lesion in the initial stage of inflammation, it moves to the liver through the bloodstream, followed by the rapid induction of an extensive range of acute-phase proteins such as CRP, fibrinogen, etc. This phenomenon opens up opportunities for researchers to study IL-6 in blood samples [20,21]. 

Our study detected higher IL-6 levels in CC patients compared to the control group with NILM. Analogous findings about increased serum IL-6 levels were reported in the Cai et al. study, which evaluated the diagnostic value of serum IL-6 in detecting CC patients [20]. Important data were observed in the Bonin-Jacob et al. study [21]. This study evaluated different levels of cytokines, including IL-6, in the exfoliated cervical cells (ECCs) and in the serum of patients regarding positive HR-HPV DNA, a majority of which did not have cervical lesions. The comparison of IL-6 levels in the serum and ECCs of HR-HPV-positive patients revealed higher levels of IL-6 in the ECCs, suggesting that IL-6 is mainly produced at the site of infection and that the immune response in the same patient changes at the systemic level [21]. Based on the reported data, the finding of elevated IL-6 values in CC patient circulation might be considered proof of significant systemic changes in the host.

For analyzing the serum pre-treatment IL-6 prognostic value for CC, we used multivariable logistic regression model. The model revealed that the chances of a patient being in the CC group is 11.4-fold higher (*p* ˂ 0.001) for increased IL-6 at a level ˃17.4 pg/mL. In the grading model, the calculated odds ratio was equal to 2.9. This means that with an increased IL-6 serum level of ˃28.7 pg/mL, the risk of being diagnosed with advanced-stage (III–IV stage) cancer increased significantly, i.e., 2.9-fold higher compared to early-stage cancer. Our findings allow us to summarize that the elevated levels of IL-6 at specified threshold values could be helpful in the prediction of CC and predictions of the advanced stage of CC.

Little data are available on serum IL-6 in gynecological tumors, especially on CC. IL-6 serum level’s correlation with metastasis and negative prognosis in ovarian cancer was mentioned by Browning et al. [22]. Another study conducted by Kampan et al. revealed IL-6 as an excellent predictive marker for distinguishing between advanced malignant and normal ovaries. Still, they analyzed only patients with advanced (III–IV stage) high-grade serous ovarian cancer (serous adenocarcinoma) but did not include patients with an early stage (I–II stage) of ovarian cancer [23]. Two studies reported a better prognosis for CC patients with lower IL-6 expression [24,25], but these studies analyzed IL-6 expression in tumor tissue but not in serum. Despite the existing differences between specimen types, these findings support those seen in our current study. Cai et al. found that serum IL-6 levels had a moderate diagnostic ability for the occurrence of CC [20], and it is quite consistent with our findings. Based on above-mentioned published data and especially on our own findings, we summarize that the elevated levels of IL-6 at specified threshold values could be helpful in the prediction of CC and predictions of advanced-stage CC.

Cancer occurrence and development are determined by tumor cells and the tumor microenvironment’s inflammatory and immune components. The major features of cancer-related inflammation include the infiltration of different cells, especially the infiltration of tumor-associated macrophages (TAMs). The presence of polypeptide messengers of inflammation was proved to be essential. One is TREM-1, which plays an important role in the occurrence and amplification of inflammatory reactions and innate immune responses. Many studies have analyzed the significance of TREM-1 in various diseases related to infection and non-infectious inflammation [26].

Researchers have intensively focused on TREM-1′s role in cancer diseases. The prognostic value of TREM-1 was analyzed in breast cancer, non-small-cell lung cancer (NSCLC), papillary thyroid cancer (PTC), etc. The recent data indicated that TREM-1 might promote tumor development and metastasis by promoting the tumor immune microenvironment, and increased TREM-1 values could be a prognostic factor for cancer outcomes and may contribute to myeloid-mediated cancer progression and immune suppression, cancer recurrence, and poor survival. Promising data are collected by investigating the prognostic impact and immunological relevance of TREM-1 expression in autoimmune diseases to identify a new strategy to improve the efficacy of immunotherapy in autoimmune diseases and in cancers as well [26]. Minimal data exist about TREM-1 related to CC.

An important task of our study was the evaluation of TREM-1 to determine threshold values and apply them to the prognosis and staging of CC. Our current study observed higher TREM-1 levels in patients with CC compared to the control group. A multivariable logistic regression model for TREM-1 regarding cervico-vaginal cytology findings determined an odds ratio value equal to 5.9. This model revealed that the chances of a patient being in the CC group were higher for increased TREM-1 at a level ˃355.6 pg/mL (*p* ˂ 0.001). However, we failed to reveal the significance of increased TREM-1 for the risk of being diagnosed with advanced-stage (III–IV stage) CC, and a possible explanation for this might be the small number of study patients.

The lack of studies on TREM1 in CC remains an actual problem for detailed comparison and analysis of study data. The most recent study by Zou et al. provided pan-cancer data analyzed by complex molecular and statistical methods to explore the abnormal expression, predictive value, and immunosuppressive role of TREM-1 in a variety of tumor analyses [26]. They demonstrated the overexpression of TREM-1 in tumors and determined the close correlation between TREM-1 and unfavorable outcomes, the infiltration of immune-suppressive cells, and immune regulation, highlighting TREM-1′s potential use as a tumor prognostic biomarker and a novel target for immunotherapy. Zou et al.’s findings were based on bladder urothelial carcinoma, breast invasive carcinoma, kidney renal clear-cell carcinoma samples, and corresponding para-carcinoma normal tissue analyses [26]. Thus, differences in study sites and sample types limit the comparability of our data. Nevertheless, the general trends of changes are similar, and the findings of the both studies do not contradict each other. According to our findings, the elevated levels of TREM-1 at specified threshold values could be helpful in the prediction of CC, but not in the prediction of the staging of CC. However, we have to admit that additional validation studies are needed.

Human and animal studies showed the significant role of LCN2 in immune and inflammatory responses, the progression of different types of human tumors, survival, and resistance to anticancer therapies via different mechanisms, such as sequestration of iron, angiogenesis, lymphangiogenesis, cell proliferation, apoptosis resistance, and cell cycle arrest [27,28]. In our previous publication, we reported that the systemic level of LCN2 was associated with chronic cervical inflammation during HPV infection, and our data supported the hypothesis that LCN2 might be important in the oncogenesis of CC. Also, we concluded that the most suitable application of LCN2 serum level measurement was for the identification of patients with advanced disease [29]. Yang et al. have analyzed LCN2 expression in breast cancer and showed the link between high LCN2 expression levels and worse survival in patients with breast cancer [27]. Villodre et al. observed that the depletion of LCN2 in inflammatory breast cancer cell lines in mouse models reduced colony formation, migration, and cancer stem cell populations in vitro and also inhibited tumor growth, skin invasion, and brain metastasis. These findings supported the idea that LCN2 promotes breast cancer aggressiveness, and modulation of LCN2 levels might serve as a new potential therapeutic target [28]. Similar data were demonstrated in CC cells; treatment with LCN2-neutralizing antibodies reduced the migration and invasion of cells that overexpressed LCN2 in humans [30]. Recent studies suggested that LCN2 could be a priority target in breast cancer and other aggressive tumors, but little is known regarding the prognostic role of LCN2 in CC. Our study observed elevated LCN2 levels in CC patients compared to the control group. Data from other studies are consistent with ours. Cymbaluk et al. provided similar information obtained from a different groups of patients with cancer. They evaluated the pattern of LCN2 expression in patients with endometrial cancer and found higher serum LCN2 concentrations in patients with endometrial cancer compared to patients with normal endometrium [31].

Using a multivariable logistic regression model, we revealed that at an LCN2 serum level ˃23,721.5 pg/mL, the chances of a patient being in the CC group was 3.45-fold higher (*p* = 0.005), and at an LCN2 serum level ˃25,640.0 pg/mL, the risk of being diagnosed with advanced-stage (III–IV stage) CC increased 4.815 fold (*p* = 0.002).

Despite the fact that the actions of LCN2 in CC have not been fully investigated, it is evident that the elevated levels of LCN2 at specified threshold values could be helpful in the prediction of CC and the advanced-stage of CC. Our findings are consistent with the study on endometrial cancer patients [31]. The lack of LCN2 studies in CC limits our ability to make deeper comparisons of our data with other studies. In addition, we would like to mention that many LCN2 studies examine it as a local rather than a systemic marker.

The small sample size limits this study, and hence, the results of the study should be interpreted with caution. The role of inflammatory markers was not separately analyzed for each stage of CC due to the small patient population in this study.

The strengths of this study are that it is a prospective study, and it uses a homogenous sample with a single tumor type. Its results have the potential to be of clinical relevance.

## 5. Conclusions

In summary, in this study, using multivariable logistic regression analyses, we provided evidence that pre-treatment serum inflammation markers IL-6, TREM-1, and LCN2 at specified levels could be used as predictors of cervical cancer, and IL-6 and LCN2 could be used as predictors of an increased chance of advanced-stage (III–IV stages) cervical cancer.

The other variables, including IFN-β, IFN-γ, IL-1β, IL-2, IL-10, IL-12p70, and TNFα, did not contribute significantly to prediction. 

Patients with cervical cancer had expressed systemic inflammation, and the expression of inflammation elevated the chance of having CC and advanced-stage disease.

## Figures and Tables

**Table 1:** Patients’ age in relation to cervico-vaginal cytology findings and stage of cervical cancer disease.

Patients Group	*n*	Age, Median [25th–75th Percentile], Years	Age Limits, Years	*p* Value
NILM	88	48 [44.0–53.75]	25–80	0.004
CC	94	55 [43.0–66.5]	22–91	
CC I stage	6	44 [32.25–56.75]	30–68	>0.05
CC II stage	41	57 [45.25–69.75]	27–91	
CC III stage	35	51 [43.0–64.0]	22–82	
CC IV stage	12	60.5 [41.0–77.5]	35–85	

CC—cervical cancer; NILM—negative for intraepithelial lesions or malignancy.

**Table 2:** Distribution of threshold values of inflammatory markers according to cervico-vaginal cytology findings.

Inflammatory Markers Predictive Value, pg/mL	Area under ROC Curve, %	Sensitivity/ Specificity, %	NILM/ CC, *n* (%)	*p* Value	CC Group OR [95% PI]
IFN-β > 79.0	58.2	86.2 35.2	57 (64.8) 81 (86.2)	0.001	3.389 [1.632–7.038]
IFN-γ > 1972.7	65.9	46.8 85.2	13 (14.8) 44 (46.8)	<0.001	5.077 [2.484–10.375]
IL-1β > 145.3	65.4	86.2 42.0	51 (58.0) 81 (86.2)	<0.001	4.52 [2.195–9.31]
IL-10 ≤ 1472.5	66.8	79.8 51.1	43 (48.9) 75 (79.8)	<0.001	4.131 [2.147–7.947]
LCN2 > 23,721.5	69.9	59.6 83.0	15 (17.0) 56 (59.6)	<0.001	7.172 [3.591–14.323]
TREM-1 > 355.6	75.0	55.3 88.6	10 (11.4) 52 (55.3)	<0.001	9.657 [4.455–20.936]
TNF-α > 17.6	90.0	96.8 81.6	16 (18.4) 91 (96.8)	<0.001	134.604 [37.742–480.06]
IL-6 > 17.4	84.6	80.6 75.9	21 (24.1) 75 (80.6)	<0.001	13.095 [6.431–26.667]

OR—odds ratio; CI—confidence interval; CC—cervical cancer; NILM—negative for intraepithelial lesions or malignancy.

**Table 3:** Multivariable binary logistic regression model for cervical cancer according to the inflammatory marker’s value.

Inflammatory Markers Predictive Value, pg/mL	CC Group OR [95% PI]	*p* Value
The model predicts correctly 81.7%, Nagelkerke coefficient of determination 0.583		
LCN2 > 23,721.5	3.448 [1.455–8.166]	0.005
TREM-1 > 355.6	5.965 [2.257–15.767]	<0.001
IL-6 > 17.4	11,432 [4.897–26.684]	<0.001
Constant of model	−2.85	<0.001

OR—odds ratio; CI—confidence interval; CC—cervical cancer.

**Table 4:** Median concentrations of investigated inflammatory markers depending on the stage of cervical cancer.

Inflammatory Marker	CC Early Stage (I–II Stage)	CC Advanced-Stage (III–IV Stage)	*p* Value
	Median [25th–75th Percentile], pg/mL		
TNF-α	60.7 [40.5–70.4]	63.4 [55.8–75.9]	0.215
IFN-β	97.2 [83.8–115.4]	95.8 [84.0–102.9]	0.402
IFN-γ	1783.05 [1711.5–2512.3]	1802.4 [1756.3–2550.1]	0.138
IL-1β	168.2 [142.5–327.5]	235.1 [169.3–370.0]	0.01
IL-2	550.5 [134.2–584.0]	290.1 [115.6–576.4]	0.072
IL-6	23.5 [14.1–44.7]	37.8 [23.4–102.8]	0.004
IL-10	1140.7 [652.6–1462.0]	1346.6 [840.0–1470.9]	0.472
IL-12p70	667.8 [151.3–701.4]	380.5 [151.3–686.4]	0.108
LCN2	12 754.6 [1279.4–32,539.8]	35,128.3 [26,558.0–40,071.3]	<0.001
TREM-1	339.4 [211.6–397.1]	391.3 [325.3–472.3]	0.029

*p* value, Mann–Whitney test; CC—cervical cancer.

**Table 5:** Distribution of threshold values of inflammatory marker’s according to the stage of cervical cancer.

Inflammatory Markers Predictive Value, pg/mL	Area under ROC Curve, %	Sensitivity/Specificity, %	CC Early Stage (I–II Stage)/ CC Advanced-Stage (III–IV Stage), n (%)	*p* Value	CC Advanced-Stage (III–IV Stage) Group OR [95% PI]
LCN2 > 25,640.0	75.4	78.7 67.4	15 (32.6) 37 (78.7)	<0.001	7.647 [3.012–19.413]
IL-6 > 28.7	67.2	73.9 56.5	20 (43.5) 34 (73.9)	0.003	3.683 [1.529–8.873]

OR—odds ratio; CI—confidence interval; CC—cervical cancer.

**Table 6:** Multivariable binary logistic regression model for advanced-stage cervical cancer (III–IV stage) according to the inflammatory marker’s value.

Inflammatory Markers Predictive Value, pg/mL	CC Advanced-Stage (III–IV Stage) Group OR [95% PI]	*p* Value
The model predicts correctly 75.0%, Nagelkerke coefficient of determination 0.351		
LCN2 > 25,640.0	4.815 [1.78–13.026]	0.002
IL-6 > 28.7	2.921 [1.06–8.045]	0.038
Constant of model	−2.292	<0.001

OR—odds ratio; CI—confidence interval; CC—cervical cancer.

## Data Availability

Data are available upon request to the corresponding author.
